# A dual-specific CRISPR-Cas nanosystem for precision therapeutic editing of liver disorders

**DOI:** 10.1038/s41392-022-01071-2

**Published:** 2022-08-12

**Authors:** Xiaojie Xu, Honglin Tang, Jiajing Guo, Huhu Xin, Yuan Ping

**Affiliations:** 1 College of Pharmaceutical Sciences, Zhejiang University, Hangzhou, 310058 China; 2 Department of Medical Oncology, Sir Run Run Shaw Hospital, School of Medicine, Zhejiang University, Hangzhou, 310058 China; 3 Liangzhu Laboratory, Zhejiang University Medical Center, 1369 West Wenyi Road, Hangzhou, 311121 China

**Keywords:** Gene delivery, Nanobiotechnology, Molecular medicine

**Dear Editor,**

The most common liver disorders, such as acute liver injury and liver fibrosis, can induce acute or chronic inflammatory responses, which in turn gradually promote the development and progression of the disorder.^1^ To date, limited therapeutic options are available for the treatment of these chronic or acute liver disorders. Recently, clustered, regularly interspaced, short palindromic repeat (CRISPR)-associated (CRISPR-Cas) systems have been harnessed as a therapeutic tool for treating genetic and non-genetic liver disorders.^2,3^ However, in vivo delivery of either Cas9-based genome editor or CasRx-based RNA editor by viral or non-viral vectors inevitably suffers from non-specific distribution upon the intravenous administration, leading to the accumulation in non-targeted organs and tissues. As a result, the unwanted editing may cause genotoxicity and serious side effects that are difficult to predict.

Herein, we report a dual liver-specific Cas-mediated DNA or RNA editing system that combines the liver-targeted delivery and liver-specific editing for the precise treatment of inflammatory liver diseases. The dual-specific editing system is designed as follows. First, a plasmid encoding the Cas editor (Cas9 or CasRx) is constructed with P3 promoter, a synthetic chimeric liver-specific promoter which is active only in the liver parenchymal cells. Second, a poly(disulfide) (PD), which has been demonstrated to be efficient in delivering Cas9 plasmid,^2^ is used to condense the plasmid to form PD/plasmid (PD/P) nanocomplexes. Finally, a layer of biomimetic macrophage membrane is coated onto the surface of PD/P nanocomplexes to afford the targeted delivery of the plasmid to the liver inflammatory lesion. Upon systemic administration, the macrophage membrane can direct the polyplexes to the liver inflammatory lesion where the transcription of the plasmid can be initiated to express the encoded editor (Cas9 or CasRx) to afford liver-specific editing, disrupting target gene or downregulating its mRNA associated with the progression of liver disorders (Fig. 1a). Although there is a small portion of Cas editors distributed in the non-targeted organs or tissues, the expression is largely limited due to the inactive transcription process controlled by P3 promoter (Fig. 1b). As such, the liver-targeted delivery and liver-specific expression of Cas editors greatly promote safe and specific DNA or RNA editing in vivo.

**Fig. 1:**
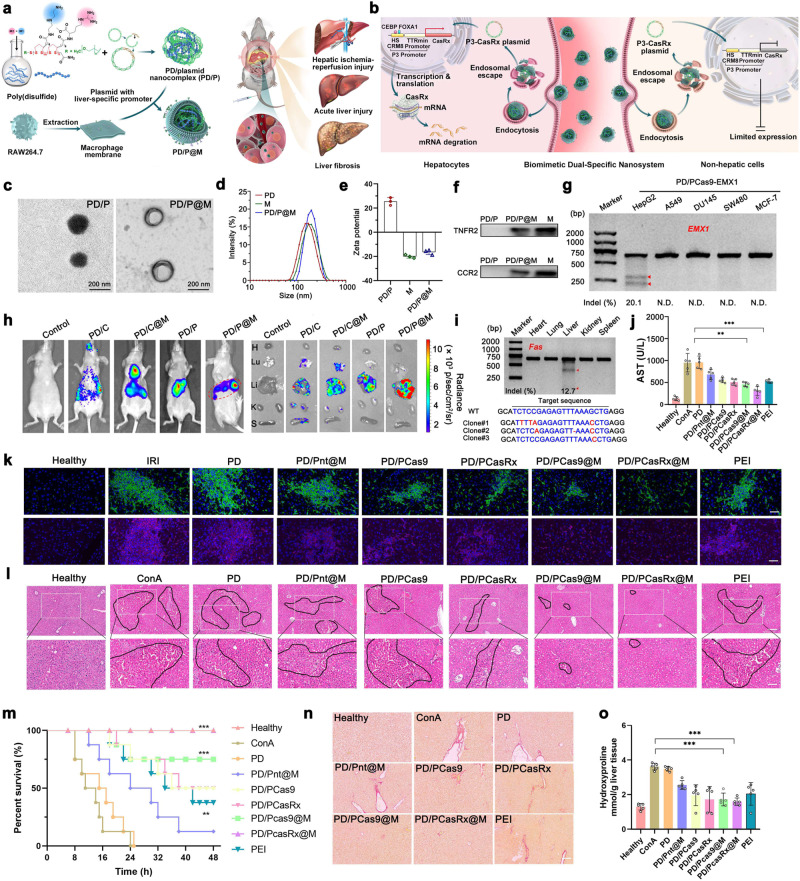
**a**, **b** Schematic illustration of the biomimetic dual-specific nanosystem for liver targeting and hepatocyte-specific RNA editing for the treatment of hepatic ischemia reperfusion, liver fibrosis and acute liver injury. **c** Representative TEM image of PD/P and PD/P@M. Scale bar: 200 nm. Particle size (**d**) and ζ potential (**e**) of M, PD/P and PD/P@M complex analyzed by dynamic light scattering (DLS). Mean ± S.D., *n* = 3. **f** Characteristic protein bands of PD, PD/P@M, and M indicated by western blot. **g** Indel mutations of *EMX1* locus detected by T7E1 assay after the indicated cells were transfected with PD/P-Cas9-*EMX1* complexes. **h** Evaluation of in vivo luciferase expression of CMV-Cas9 (C) or P3-Cas9 (P) plasmid with luciferase tag in BALB/c mice and dislodged organs. H, heart; Lu lung; Li, liver; K, kidney; S, spleen. **i** Indel mutations of *Fas* locus in different organs as detected by T7E1 assay and Sanger sequencing of the liver tissue. **j** The serum AST level after the indicated treatment of mice with hepatic ischemia-reperfusion injury. **k** Immunofluorescence staining of the liver samples after the indicated treatment. Anti-F4/80 antibody (green) was used for monocyte/macrophage marker, anti-Ly6G antibody (red) was used for neutrophils marker, and DAPI (blue) for nuclear staining of liver tissues from each group. Scale bar: 50 µm. **l** The H&E staining of the liver from mice with acute liver injury after the indicated treatment. The region of the black line denotes the accumulation of blood cells. Scar bar, top panel, 200 µm; scar bar, lower panel, 100 µm. **m** Survival rates of mice after the treatment. (*n* = 8), Log-rank test (Mantel-cox test). **n** Liver sections were stained for sirius red to assess the degreecollagen deposition and fibrillar collagen. Scale bar: 200 µm. **o** Hepatic hydroxyproline from the liver fibrosis mice administered with complexes. All of the data analyzes as mean ± S.D., *n* = 5. One-way ANOVA with a Tukey’s post-hoc test. *P < 0.05; **P < 0.01 ***P < 0.001

Firstly, the most efficient PD for Cas9 plasmid delivery was obtained by polymerizing two monomers containing cationic diethylenetriamine and guanidyl moieties.^4^ The macrophage membrane-coated polyplexes (PD/P@M) were further developed by coating the macrophage membrane from Raw264.7 cells onto the exofacial surface of PD/P via a well-established extrusion method.^5^ The morphology of PD/P@M was characterized by transmission electron microscopy (TEM), where a thin layer of membrane (~ 9 nm) was observed (Fig. 1c). Whereas the size distribution of PD/P@M was in a similar range as uncoated polyplexes (Fig. 1d), the ζ potential reversed from positive to negative after the membrane coating (Fig. 1e). We found the typical protein profiles from macrophages were presented in PD/P@M (Supplementary Fig. 1a), and the key antigens from the macrophage membrane, including CCR2 (a receptor for monocyte chemoattractant protein-1) and TNFR2 (tumor necrosis factor receptor 2) that play important roles in inflammatory homing and anti-inflammatory effects, were well reserved (Fig. 1f). It was obvious that DNA migration was completely inhibited at the N/P ratio of 2 (Supplementary Fig. 1b), and the membrane coating did not affect polymer/DNA complexation (Supplementary Fig. 1c). To further investigate the endosomal escape, we observed that a large portion of red fluorescence (DNA) spread out from the green fluorescence (lysosome), suggesting the intracellular release of the plasmid from endosomes (Supplementary Fig. 1d). Moreover, the western blot analysis of Cas9flag or CasRxflag protein expression after the transfection indicated the plasmids trafficking from the cytoplasm to the nucleus were translated into CasRx or Cas9 (Supplementary Fig. 1e). As compared with other types of cells, the transfection of HepG2 cells by PD/P3 complexes showed much stronger green fluorescent protein (GFP) expression, indicating high hepatocyte specificity (Supplementary Fig. 1f). The intracellular delivery of P3-Cas9-EGFP plasmid mediated by PD in AML12 cells reached a comparable efficiency as transfecting CMV-Cas9-GFP plasmid (Supplementary Fig. 1g). Furthermore, the excellent biomimetic tropism ability of exofacial macrophage membrane to target inflammatory tissues was also confirmed after the systemic delivery in vivo (Supplementary Fig. 1h).

We evaluated whether P3 promoter that includes a hepatocyte-specific *cis*-regulatory module (HS-CRM8) and a minimal transthyretin (TTRmin) promoter can induce the gene expression of encoded Cas9 or CasRx in a liver-specific manner. To this end, we transfected the plasmid into a range of human cell lines that derived from different organs or tissues, and the T7 endonuclease I (T7EI) digestion assays showed that the cleaved bands were only detected in HepG2 cells, with an indel frequency of 20.1%; however, the cleaved bands were almost undetectable in other types of cell lines (including A549, DU145, SW480, and MCF-7), suggesting the high specificity of transcription driven by P3 promoter (Fig. 1g). To further validate the liver specificity in vivo, the systemic transfection of P3-driven Cas9 plasmid simultaneously encoding a luciferase tag, delivered by PD/P@M, further confirmed the exclusive, strong luciferase expression in the liver areas, with minimal non-specific luciferase expression in other organs, such as lung and kidney (Fig. 1h). In addition, T7EI assay also demonstrated the exceptional specificity of genome editing in the liver of mouse with liver fibrosis (Fig. 1i). These results suggested the excellent biomimetic tropism ability of exofacial macrophage membrane to target inflammatory tissues and liver-specific expression driven by P3 promoter after the systemic delivery in vivo.

The disruption of arachidonate 12-lipoxygenase (*Alox12*) gene or the direct degradation of *Alox12* mRNA to downregulate its protein level to block 12-HETE production represents a viable approach to prevent and treat ischemia-reperfusion (IR)-induced liver damage.^6^ Likewise, the inhibition of *Fas* gene expression could protect mice from fulminant hepatic failure, and the direct disruption of *Fas* gene could prevent the progress of chronic hepatitis, thereby alleviating the symptoms of liver fibrosis.^7^ The ability of PD/P and PD/P@M to mediate liver-specific genome editing or RNA editing indicated the downregulation of relevant gene expression in vitro (Supplementary Fig. 2). Further, *Alox12* knockdown in the liver contributes to the prevention and attenuation of ischemia reperfusion-induced injury. The disruption of DNA of *Alox12* mediated by PD/P@M could decrease its protein level in vivo (Supplementary Fig. 3e), and resulted in the change of protein expression of BCL2 (B-cell lymphoma-2, anti-apoptosis protein), BAX (BCL2-associated X, pro-apoptotic protein), and C-CASP3 (cleaved caspase-3, pro-apoptotic protein) in the mouse liver after the indicated treatment. Additionally, anti-F4/80 antibody (green) and anti-Ly6G antibody (red) that were used to indicate monocyte/macrophage cells and neutrophils suggested the inflammatory cell phenotypes reduced after the treatment (Fig. 1k). As the indicator of liver functions, the level of aspartate aminotransferase (AST) and alanine aminotransferase (ALT) became much lower after the treatment with PD/PCas9@M or PD/PCasRx@M (Fig. 1j and Supplementary Fig. 3h). The tumor necrosis factor-α (TNF-α) and interferon-gamma (IFN-γ) showed the lower level (Supplementary Fig. 3i, j), indicating the alleviation of inflammatory responses. These results collectively suggested that the dual liver-specific editing system was effective for the treatment of the hepatic ischemia-reperfusion injury (Supplementary Fig. 3).

To evaluate the therapeutic effectiveness for concanavalin A (ConA)-induced acute liver injury by PD/P@M, we first evaluated the disruption of *Fas* locus, mRNA and protein downregulation after the treatment (Supplementary Fig. 4b–f). The representative liver and H&E staining histological images suggested that the treatment with PD/PCas9@M or PD/PCasRx@M could effectively reduce hyperemia (Supplementary Fig. 4g and Fig. 1l), which was further confirmed by blood biochemistry analysis (Supplementary Fig. 4). The treatment therefore effectively prolonged the survival time of the model mice (Fig. 1m). Furthermore, we evaluated whether the dual liver-specific editing nanosystem was effective in treating liver fibrosis. We first noticed the disruption of genomic DNA of *Fas* and the downregulation of mRNA after the treatment (Supplementary Fig. 5). The immunohistochemical analysis of α smooth muscle actin (α-SMA), Sirius red staining and Masson staining of the liver slides validated the amelioration of the hepatic inflammation and fibrosis (Supplementary Fig. 5 and Fig. 1n), in which PD/PCas9@M and PD/PCasRx@M exerted the inhibitory effect of liver fibrosis. The indicators of liver fibrosis, including hydroxyproline and procollagen III, were also downregulated after the treatment (Fig. 1o and Supplementary Fig. 5i), accompanied by the decreased level of AST, ALT and TNF-α (Supplementary Fig. 5). The percentage of Cas9 and CasRx-positive cells in the liver were determined by fluorescence activated cell sorting (FACS), and we found that the number of GFP-positive cells induced by PD/PCas9@M or PD/PCasRX@M reached 14.5% or 15.1%, respectively (Supplementary Fig. 6a). The onset of transgene expression of Cas9 or CasRx mediated by PD/P@M was around 8 h (Supplementary Fig. 6b, 6c). After the systemic administration of PD/P@M, the gene expression could persist in the liver for about 21 day (Supplementary Fig. 6d). Notably, the delivery system exhibited good biocompatibility and was generally safe for in vivo delivery (Supplementary Fig. 7). Altogether, these results demonstrated that the in vivo delivery of the dual liver-specific editing nanosystem rescued the mice from acute liver injury and chronic hepatitis in a safe and efficient manner.

In conclusion, we developed a dual liver-specific CRISPR editing nanosystem for the precision therapy of inflammatory liver disorders. The systemic delivery of liver-specific Cas9 or CasRx editors by PD/P@M showed high liver specificity in vivo by avoiding unwanted editing in non-hepatic tissues, thereby precisely modulating inflammation-related signaling pathways by means of genome or RNA editing, without any observable side effects. Notably, we found liver-specific RNA editing was generally more efficacious over genome DNA editing in terms of therapeutic efficacy, and recommended CasRx-mediated RNA therapeutic editing for treating non-genetic inflammatory liver disorders.

## Supplementary information

Supplemental Material

## Data Availability

All data and materials are available on request.
