# Supplementary material for: A dual-specific CRISPR-Cas nanosystem for precision therapeutic editing of liver disorders
Source: Signal Transduct Target Ther. 2022 Aug 12;7:269. doi: 10.1038/s41392-022-01071-2 (PMC9372082; doi:10.1038/s41392-022-01071-2)
Supplement: Supplementary file 1 — Supplemental Material [file 41392_2022_1071_MOESM1_ESM.docx]

**Supporting Information**

**A Dual-Specific CRISPR-Cas Nanosystem for Precision Therapeutic Editing of Liver Disorders**

Xiaojie Xu ^a,#^, Honglin Tang ^a,c,#^, Jiajing Guo ^a^, Huhu Xin ^a^, Yuan Ping ^a,b*^

^a^ College of Pharmaceutical Sciences, Zhejiang University, Hangzhou 310058, China

^b^ Liangzhu Laboratory, Zhejiang University Medical Center, 1369 West Wenyi Road, Hangzhou 311121, China

^c^ Department of Medical Oncology, Sir Run Run Shaw Hospital, School of Medicine, Zhejiang University, Hangzhou 310058, China

^#^ The authors contribute equally to this work.

^*^ Corresponding author: [pingy@zju.edu.cn](mailto:pingy@zju.edu.cn)

**This file includes:**

Materials and Methods

Supplementary Figure S1-S9

Supplemental Tables S1-S4

**Materials and Methods**

**Materials**

Lipoic acid, 1,1'-carbonyldiimidazole (CDI), diethylenetriamine, L-Arginine methyl ester dihydrochloride, ICG-NHS, N-acetyl-L-cysteine methyl ester, and TEOA purchased from Sigma-Aldrich (USA), Lipofectamine 3000 (Lipo 3000) was purchased from Thermo Fisher Scientific (USA). Dulbecco’s modified Eagle’s medium (DMEM), RPMI 1640 medium, L15 medium and DMEM-F12 medium were purchased from Sigma-Aldrich (USA). The protein release kit (BCA kit) was purchased from Beyotime (China). Fetal bovine serum (FBS) was purchased from ZhejiangTianhang Biotechnology Co.Ltd. (China). T7 endonuclease I (T7EI) enzyme was purchased from GenScript (USA). The FastPure® cell/tissue DNA Isolation Mini Kit and FastPure® Gel DNA Extraction Mini Kit were purchased from Vazyme Biotech Co., Ltd (China). Ultrapure water was obtained from a Milli-Q system. D-luciferin-K^+^ salt bioluminescent substrate was purchased from Sigma-Aldrich (USA). Size and zeta potentials of the polyplexes were characterized by Malvern Nano ZS90. Transmission electron microscopy (TEM) was carried out on a transmission electron microscope (HT7700, Hitachi, Japan). The luciferase intensity was evaluated by vivo imaging system (IVIS® Spectrum, PerkinElmer). Confocal microscopy was performed on a Carl Zeiss, LSM-880 (Germany). Antibodies against Alox12 (PA5-78760) and Fas (MA1-7622) were obtained from Thermo Fisher Scientific (USA). Anti-BCL2 (catalog no. 3498), anti-BAX (catalog no. 2772), anti-c-CASP3 (catalog no. 9664) and anti-GAPDH (catalog no. 2118) antibodies were obtained from Cell Signaling Technology (USA).

**Cell culture and transfection**

HepG2, MCF-7 and RAW264.7 cell lines were cultured in Dulbecco’s Eagle Medium with high glucose. A549, DU145 and cells were cultured in RPMI 1640 with high glucose. SW480 cells were cultured in L15 Medium. AML12 cells were cultured in DMEM-F12 Medium. All of the above medium was supplemented with 10% fetal bovine serum, and cultured the indicated cells at 37 ℃ with 5% CO_2_. For the transfection steps, cells were transplanted in 48-well plates at the density of 80%. The transfected mixture was added into the plates and incubated for 6 h with serum-free medium, and then changed to fresh 10% fetal bovine serum culture medium without antibiotics. The transfection images were visualized by the fluorescence microscope at 48 h post-transfection. The cells were harvested at 48 h transfection for the measurement of the luciferase activities and other metrics.

**Synthesis and characterization of PD and PD/P@M**

The polymerization of poly(disulfide)s (termed as PD) has two monomers containing cationic diethylenetriamine (DET) moieties and guanidyl groups respectively. Ring-opening polymerization of two monomers was initiated by thiolated PEG or N-acetyl-L-cysteine methyl ester ^1^. PD was synthesized as previously reported. In detail, the monomer 1 (M1) was synthesized as described in the following steps: commercially available lipoic acid (4.12 g, 20 mmol) and CDI (4.28 g, 26.4 mmol) were dissolved in dry DCM (100 ml), which was dropwise added into the solutions of diethylenetriamine 2 (DET, 16 g, 155 mmol) dissolved in dry DCM (30 ml). The reaction mixture was stirred for 1 h at 0 °C and restored at room temperature for 1 h. The brine (3 × 100 ml) was used to wash the terminal products. M1 (1.9 g, 50%) was obtained after the organic layer dried and concentrated. The monomer 2 (M2) was synthesis as described in the following steps: firstly, lipoic acid (4.12 g, 20 mmol) and CDI (3.24 g, 20 mmol) were added in anhydrous DMF (50 ml), following by stirring at room temperature for 2 h. The above mixture was added into the solutions of methyl L-arginine methyl ester dihydrochloride (2.61 g, 10 mmol) and DIEA (1.74 µl, 10 mmol) in anhydrous DMF (40 ml), following by stirring at room temperature for 3.5 h. The mixture solution was added dropwise into Et2O (200 ml) for precipitation. The precipitate was collected after the centrifugation (2 min, 3000 rpm). The obtained yellow oil was washed with MeOH/ Et2O mixture (1:2, 50 × 100 ml) for three times. M2 was obtained by the purified with flash silica gel column chromatography (DCM/MeOH = 50:1). The polymerization of DET-CPDs was performed by M1 and M2 (1:2 in ratio, 2.4 mM in total). The solution of initiator 6 (0.03 mM) in 2 ml TEOA buffer (1.0 M, pH = 7) was added in solution of M1 and M2 in 10 ml TEOA buffer and then the reaction mixture stirred vigorously for a while in N2 atmosphere. For polymerization, the sample kept at room temperature with vigorous agitation, after 1.5 h, the polymerization mixture was taken and quenched in a solution of terminator 5 (12 mM) in water, and then the mixture was dialyzed in water for 7 days, obtaining the poly(disulfide) named DET-CPDs (PD).

RAW264.7 cells were collected for membrane extraction. And the membrane was extracted by the Membrane and Cytosol Protein Extraction Kit (Beyotime Biotechnology, China, #P0033), all of the steps was performed according to the manufacturer`s recommended protocol. The bicinchoninic acid assay (BCA) protein assay was used to analyze the total protein content in the macrophage membrane. The collected membrane was stored at -80 °C for further study.

The membrane coating PD/P (PD/P@M, Plasmid termed as P) was performed by fusing RAW264.7 membrane with PD/P via a mini-extruder for 30 times. Before the extrusion steps, substantial sonication was performed to the mixture by using the bath sonicator at 40 kHz and 100 W for 2 min. The uncoated membrane was removed by centrifuged at 3000 × g for 30 min. After then, the mixture was centrifuged at 12000 × g for 5 min to obtain the precipitate ^2,3^.TEM image of M (membrane), PD, and PD/P@M were performed on a transmission electron microscope (HT7700, Hitachi, Japan). Size and zeta potentials of M (membrane), PD, and PD/P@M were characterized by DLS at 25 ℃ (Nano ZS90, Malvern Panalytical, UK).

**Liver-specific expression plasmid construction**

CMV-Cas9-GFP-U6-sgRNA and pBS-CAG-CasRx-P2A-GFP-pA plasmids were constructed by in-fusion cloning strategy for further engineered ^4^. All of the fragments were amplified by PCR. To construct P3-Cas9-GFP-U6-sgRNA and pBS-P3-CasRx-P2A-GFP-pA plasmid, the P3 promoter sequence was synthesized by Youkang Biotechnology and inserted into the plasmid of CMV-Cas9-GFP-U6-sgRNA and pBS-CAG-CasRx-P2A-GFP-pA by replacing CMV promoter and CAG promoter through restriction site of Hind III and SaII, and EcoRI and SaII, respectively ^5-8^. U6-sgRNA fragment was added to the plasmid by the restriction site of SaII. P3-Cas9-GFP-luci-U6-sgRNA plasmid was constructed similarly by molecular cloning methods.

**Gel retardation assays**

The condensable ability of polymer to DNA was detected by gel electrophoresis. The polymer was condensed with P3-CasRx-GFP plasmid at different N/P ratio to from the complex and incubated at room temperature for 30 min. Then, the complex was added in the 2 % agarose gels, and run in TAE buffer (40 mM Trisacetate, 1 mM EDTA) at 120 v for 40 min in a JUNYI Electrophoresis Equipment (JY300, Beijing Junyi-Dongfang, China). Subsequently, Gel Documentation System (c150, Azure Biosystems, USA) was used to visualize DNA bands for further analysis.

**Cellular uptake of PD/P complexes**

The cellular uptake of PD/P complexes was measured as previously described ^9^. Briefly, P3-Cas9-GFP-U6-sgRNA plasmid was labeled by IT rhodamine in red and then complexed with the polymer at indicated ratio. The complex of PD/P in serum-free culture medium was added to the cultured AML12 cells for 1 h, 3 h, and 6 h. After transfection, AML12 cell nucleus was stained with Hoechst 33342 in blue, and the lysosome was stained with lysotracker green DND-26. The confocal images of cells transfected with PD/P complex was visualized by confocal laser scanning microscopy (CLSM, LSM880) at 1 h, 3 h and 6 h, respectively.

**Quantitative reverse transcription PCR (qRT-PCR)**

The knockdown efficiency was assessed by qRT-PCR. Firstly, the total RNA was extracted by RNA Isolator Total RNA Extraction Reagent (Vazyme Biotech Co., Ltd). The extracted RNA was used as the template to transcribe into complementary DNA (cDNA) by reverse transcriptase. For the further analysis, the cDNA was diluted to a suitable dilution and served as templates for qRT-PCR reactions. Two-step qRT-PCR incorporating SYBR Green qRT-PCR methodology was performed (Yeasen Biotech Co., Ltd). PCR primers for the qPCR step were retrieved from PrimerBank (<http://pgamgh.harvard.edu/primerbank/>) and the detailed sequence was displayed in table S4.

**Western blot**

The proteins were extracted from the cell lines and liver tissues by RIPA lysis buffer containing 1% protease inhibitor. The concentration of protein was measured by the BCA kit (Thermo Fisher) and adjusted equally with RIPA lysis buffer. Protein lysates were separated by SDS polyacrylamide gel electrophoresis (SDS-PAGE) at 80 V of concentrated gel and 120 V of separation glue. The protein gel was transferred onto PVDF membrane according to the standard procedure. The PVDF membrane was blocked with 5% BSA for 1.5 h and incubated with the primary antibodies against Alox12, Fas, BCL2, BAX, C-CASP3, and GAPDH (1:2000, Santa Cruz) at 4 °C overnight.

**T7EI assays and deep-sequencing analysis**

The indel frequency of the target genomic loci was assessed by T7EI assay. The DNA from transfected cells and liver tissues were extracted by using DNeasy Blood & Tissue Kit (Vazyme, China). The target regions were amplified by PCR by using QIA quick PCR Purification Kit (Vazyme, China), and the detailed primers were displayed in Table S3. The 200 ng PCR products were used to perform T7EI assay according to the recommended protocol. Products were analyzed by agarose gel electrophoresis (2% agarose gel) and visualized by gel documentation system (c150, Azure Biosystems, USA). Finally, the quantification of gray level of DNA bands were measured by using Image J. The indel frequency analysis was calculated by the formula: [1-(1-fraction cleaved)1/2] × 100%, in which the fraction cleaved = the sum of each digested band intensity / (the sum of each digested band intensity + undigested band intensity). The PCR products were further quantified by deep-sequencing assay to obtain the more precision indel frequency. All of the deep sequencing results were performed in Fig. S7.

**Immunofluorescence staining**

The collected liver slides were fixed with paraformaldehyde (4%) for 10 min, washed with PBST (PBS with 0.2% Triton) for two times, and then incubated for 15min. After washing, the slides were blocked for 1 h by the buffer containing 5% horse serum and 0.3% Triton in PBS. Subsequently, the liver sections were incubated with block buffer containing the diluted antibodies of anti-mouse F4/80, Ly6G and α-smooth muscle actin (α-SMA) overnight at 4 °C. After washed twice with PBS, the sections were stained with Alexa Fluor 488 or 594-goat anti-rabbit IgG for 1 h, and then incubated with DAPI for 20 min and observed by using the laser scanning confocal microscope (LSCM).

**Enzyme-linked immunosorbent assay (ELISA).**

The obtained liver of mice was ground after the addition of RIPA lysis buffer, followed by centrifuging at 20000 g for 20 min at 4°C. The supernatants of lysates were collected and used for further analysis. Serum was collected by centrifugation of blood at 2000g for 15 min at 4 °C. After a series of dilutions, the cytokines (TNF-α, IL-6, IFN-γ) and specific proteins (hydroxyproline and procollagen III) were detected by ELISA in accordance with the manufacturer’s instruction.

**Animals**

C57BL/6 mice (16-20 g, 5-6 weeks old) were purchased from Center for Disease Control and Prevention (Zhejiang, China). All mice were acclimated to the environment for one week. The mice were housed in the Laboratory in Animals Centre in Zhejiang University, and were housed under standard conditions of care. The light/dark cycle was 12-h light/12-h dark in a pathogen-free environment. All animal treatments or procedures were approved by the Laboratory Animal welfare and Ethics Committee of Zhejiang University.

**Protection of the hepatic ischemia-reperfusion injury (IRI) in mice**

The C57BL/6 mice (6-8 weeks) mice were randomly divided into to six groups including healthy, IRI, PD, PD/Pnt@M, PD/PCas9, PD/PCasRx, PD/Pcas9@M, PD/PCasRx@M, PEI. In order to evaluate the protective effects of our designed nanosystem, the mice were receiving the indicated treatment at day 1, day 3, and day 5, respectively. The PD/PCasRx@M complex were composed of 30 µg plasmid and 150 µL PD/Pnt@M (1.5 µg/µL). The IRI model was established at day 7, the C57BL/6 mice in IRI, PD, PD/Pnt@M, PD/PCas9, PD/PCasRx, PD/Pcas9@M, PD/PCasRx@M, PEI group were anesthetized with isoflurane, and then fixed in supine position on the surgical table. After the abdomen were depilated and disinfected, the mice were performed to exposure the liver by midline laparotomy. The left and median liver lobes were blocked with a microvascular clamp to occlude blood supply for 60 min, followed with reperfusion by removing the clamp. Bleaching of the ischemic liver lobes indicated the success of ischemia operation. The abdomen was sealed with sterile medical silk suture as previously described ^10^. Liver tissues and blood were obtained for further analysis at 24 h after reperfusion.

**Therapy of the concanavalin-A (ConA) induced hepatic fibrosis in mice**

To establish the ConA-induced hepatic fibrosis mice model, the C57BL/6 mice (6-8 weeks) were intravenously injected with the Con-A (8 mg/kg) solutions weekly for 5 weeks. The Con-A-treated mice were randomly divided into Con-A, PD, PD/Pnt@M, PD/PCas9, PD/PCasRx, PD/Pcas9@M and PD/PCasRx@M, PEI groups and administrated with the indicated nanoparticles or PBS weekly for 4 weeks. For further analysis and therapeutic evaluation, the mice were sacrificed to harvest the liver and blood at day 50. The T7EI assay, T-A cloning, deep sequencing, qRT-PCR, western blot and ELISA were performed as described previously.

**Protection of the ConA induced fulminant hepatic failure in mice**

The C57BL/6 mice (6-8 weeks) mice were randomly divided into to six groups including healthy, ConA, PD, PD/Pnt@M, PD/PCas9, PD/PCasRx, PD/Pcas9@M, PD/PCasRx@M, PEI. In order to evaluate the protective effects of our designed nanosystem, the mice were receiving the indicated treatment at day 1, day 3, day 5 and day 7, respectively. The PD/PCasRx@M complex were composed of 30 µg plasmid and 150 µL PD/Pnt@M (1.5 µg/µL). To establish the ConA induced fulminant hepatic failure model, the C57BL/6 mice in ConA, PD, PD/PCas9, PD/PCasRx, PD/Pnt@M, PD/Pcas9@M, PD/PCasRx@M, PEI group were injected with Con-A (suspended in 100µL of pyrogen-free saline, 17 mg/kg) intravenously *via* tail-vein injection at day 7. 48 h after Con-A injection the the mice were sacrificed to harvest the liver and blood. The levels of ALT and AST in serum were detected and the obtained liver sections were stained with H&E. Then, the T7EI assay, T-A cloning, deep sequencing and qRT-PCR were performed, as described previously.

***In vivo* toxicity assessment.**

The major organs and blood samples were collected from the above-mentioned groups. The plasma was harvested by centrifuged at 2000 g for 15 min at 4 ºC to text the level of total protein, albumin, globulin, alanine aminotransferase (ALT), aspartate aminotransferase (AST), AST/ALT, UA (uric acid) and BUN (blood urea nitrogen) for analysis of liver and kidney functional profiles. Histological changes were evaluated by H&E-stained major organs. The major organs of heart, liver, kidney, lung, and spleen were collected and fixed with paraformaldehyde (4% in PBS), followed by the dehydration and staining with hematoxylin-eosin (H&E).

**Statistical analysis**

All statistical analyses in this paper were performed using GraphPad Prism 8.0. All results were expressed by the mean ± standard deviation (S.D.). Biological replicates were expressed in all experiments unless otherwise stated. The Student’s t-test and analysis of variance (ANOVA) were used for statistical significance analysis in this paper. Unpaired two-tailed Students’ t-test was used for comparison of two groups. Survival benefit was determined using a log-rank test. P < 0.05 was considered significant. (* p < 0.05, ** p < 0.01, *** p < 0.001).

**Reference**

1. Guo, J. et al. Rational design of poly(disulfide)s as a universal platform for delivery of CRISPR-Cas9 machineries toward therapeutic genome editing. *ACS Cent. Sci.* **7**, 990-1000 (2021).
2. Gao, C. et al. Treatment of atherosclerosis by macrophage-biomimetic nanoparticles via targeted pharmacotherapy and sequestration of proinflammatory cytokines. *Nat. Commun.* **11**, 2622 (2020).
3. Zhang, Y. et al. Macrophage-membrane-coated nanoparticles for tumor-targeted chemotherapy. *Nano Lett.* **18**, 1908-1915 (2018).
4. Chen, X., Chen, Y., Xin, H., Wan, T. & Ping, Y. Near-infrared optogenetic engineering of photothermal nanoCRISPR for programmable genome editing. *Proc. Natl. Acad. Sci. U. S. A.* **117**, 2395-2405 (2020).
5. Chuah, M. K. et al. Liver-specific transcriptional modules identified by genome-wide in silico analysis enable efficient gene therapy in mice and non-human primates. *Mol. Ther.* **22**, 1605-1613 (2014).
6. Viecelli HM. et al. Treatment of phenylketonuria using minicircle-based naked-DNA gene transfer to murine liver. Hepatology. 60,1035-1043 (2014).
7. Nair N. et al. Computationally designed liver-specific transcriptional modules and hyperactive factor IX improve hepatic gene therapy. Blood. 123, 3195-3199 (2014).
8. Singh K. et al. Efficient in vivo liver-directed gene editing using CRISPR/Cas9. Mol Ther. 26, 1241-1254 (2018).
9. Tang, H. et al. Reprogramming the tumor microenvironment through second-near-infrared-window photothermal genome editing of PD-L1 mediated by supramolecular gold nanorods for enhanced cancer immunotherapy. *Adv. Mater.* **33**, e2006003 (2021).
10. Ni, D. et al. Ceria nanoparticles meet hepatic ischemia-reperfusion injury: The perfect imperfection. *Adv. Mater.* **31**, e1902956 (2019).


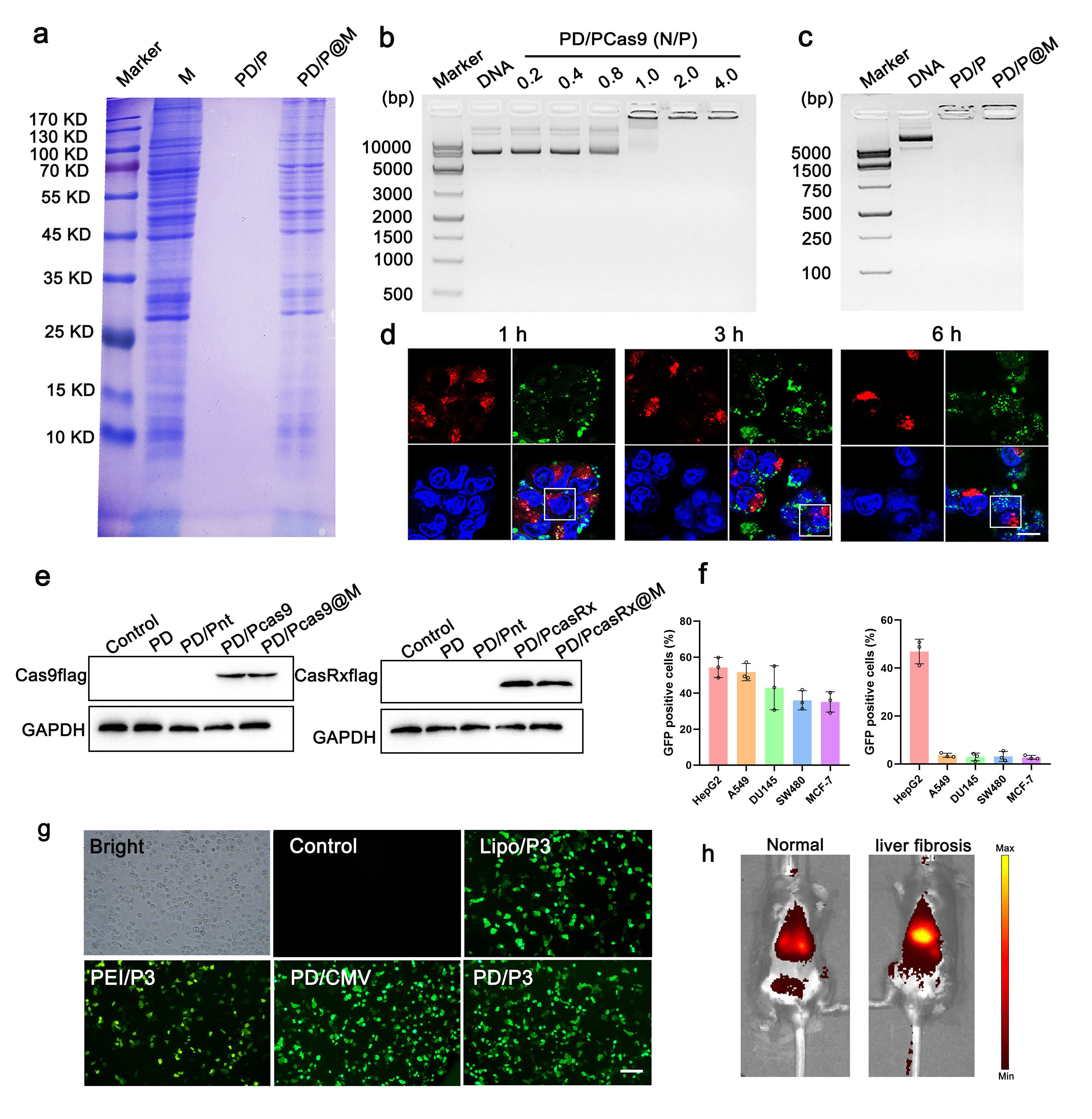


Fig. S1. (a) SDS-PAGE analysis of M (RAW membrane), PD/P (PD complex with plasmid), PD/P@M ((PD complex with plasmid and coated on the membrane). Agarose gel electrophoresis assay of PD with Cas9 plasmid at different N/P ratio (b) and PD/P complexes and PD/P@M at N/P ratio of 2 (c). (d) Confocal images of cells transfected with PD complex with P3 driven plasmid (PD/P) for different time length. The AML12 cell nucleus was stained with Hoechst 33342, the lysosome was stained with lysotracker green DND-26, and plasmid was stained with Label IT rhodamine. Scale bar, 10 μm. (e) Western blot analysis of Cas9flad or CasRxflag protein expression after the indicated treatment. (f) GFP expression of HepG2, A549, DU145, SW480 and MCF-7 cells transfected with PD/CMV-Cas9-GFP (left) and PD/P3-Cas9-GFP (right) complexes at 48 h. Data represent mean ± S.D. (n = 3). (g) GFP expression in AML12 cells mediated by P3-Cas9-GFP plasmid. Lipo- and PEI- medicated transfection were used as positive controls of PD-medicated transfection. The transfection of CMV-Cas9-GFP plasmid medicated by PD (PD/CMV) was used as positive controls of P3-Cas9-GFP plasmid, and cells without any treatment were used as negative controls. Scale bar, 100 μm. (h) Biodistribution of PD/Pnt@M in normal or liver fibrosis (increased inflammation) mice. The membrane of PD/Pnt@M was labeled by DiI and the imaging of mice received tail-vein injection of PD/Pnt@M after 48h.


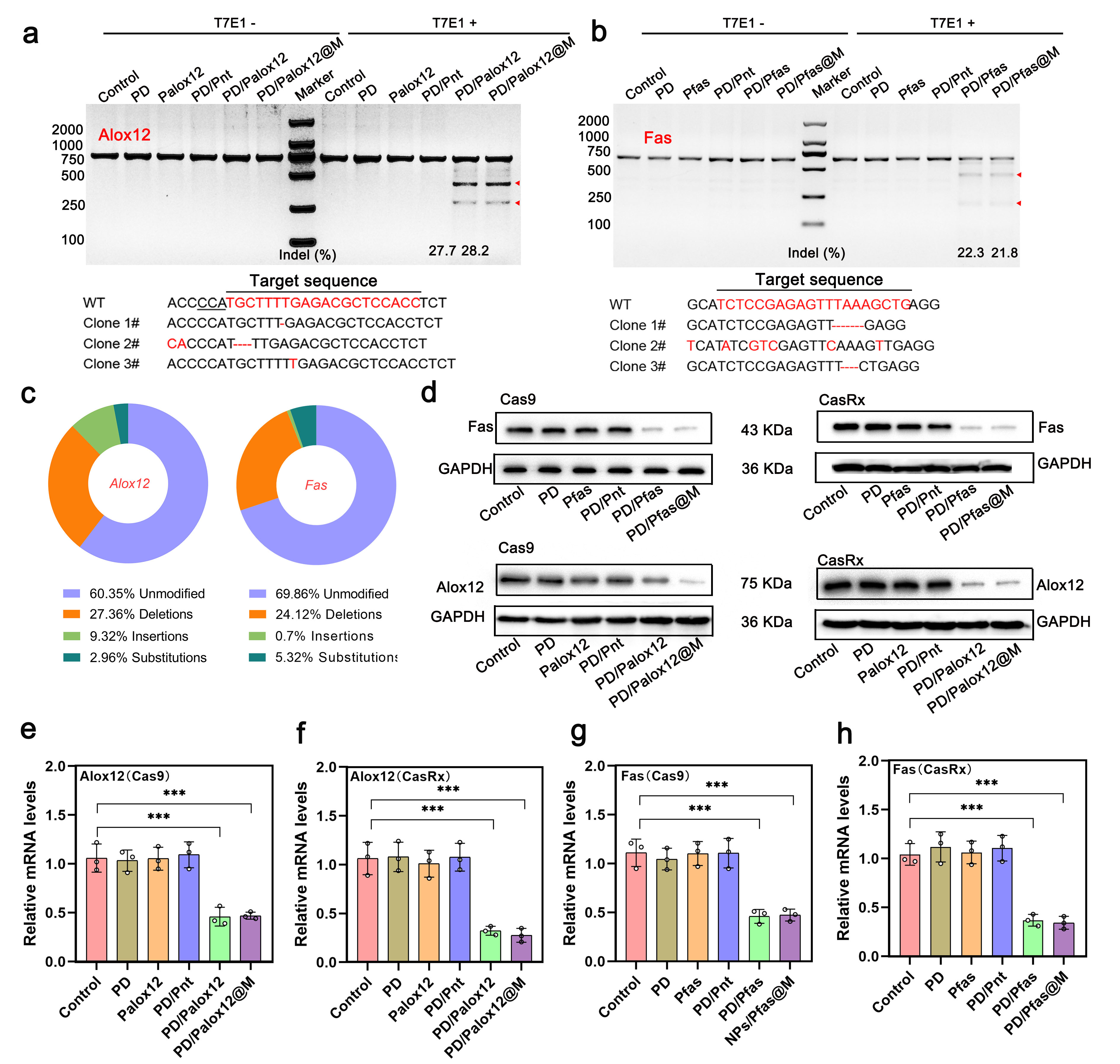


Fig. S2. Specific knockdown of *Alox12* and *Fas* *in vitro*. (a) Indel mutations of *Alox12* locus detected by T7E1 assay (upper) and Sanger sequencing (lower). AML12 cells were transfected with PD/PCas9 complexes and the indel mutations were evaluated 48 h after the transfection. (b) Genomic disruption of *Fas* after the same treatment as analyzed by T7E1 (upper), and Sanger sequencing of TA-cloning (lower). (c) Deep-sequencing analysis of mutation frequency at *Alox12* and *Fas* locus. The mutation frequency was analyzed through a single deep-sequencing library prepared from genomic DNA pooled. (d) Western blot analysis of Fas or Alox12 protein expression after the indicated transfection in AML12 cells. Relative levels of Alox12 mRNA (e,f) or Fas mRNA (g,h) after indicated transfection. Palox12, Pfas, and Pnt, denote for Cas9 or CasRx plasmid driven by a P3 promoter targeting *alox12* locus, *Fas* locus, and non-sense locus. All of the data were performed with Mean ± S.D., n = 3. One-way ANOVA with a Tukey’s post-hoc test. *P < 0.05; **P < 0.01 ***P < 0.001 vs. control.


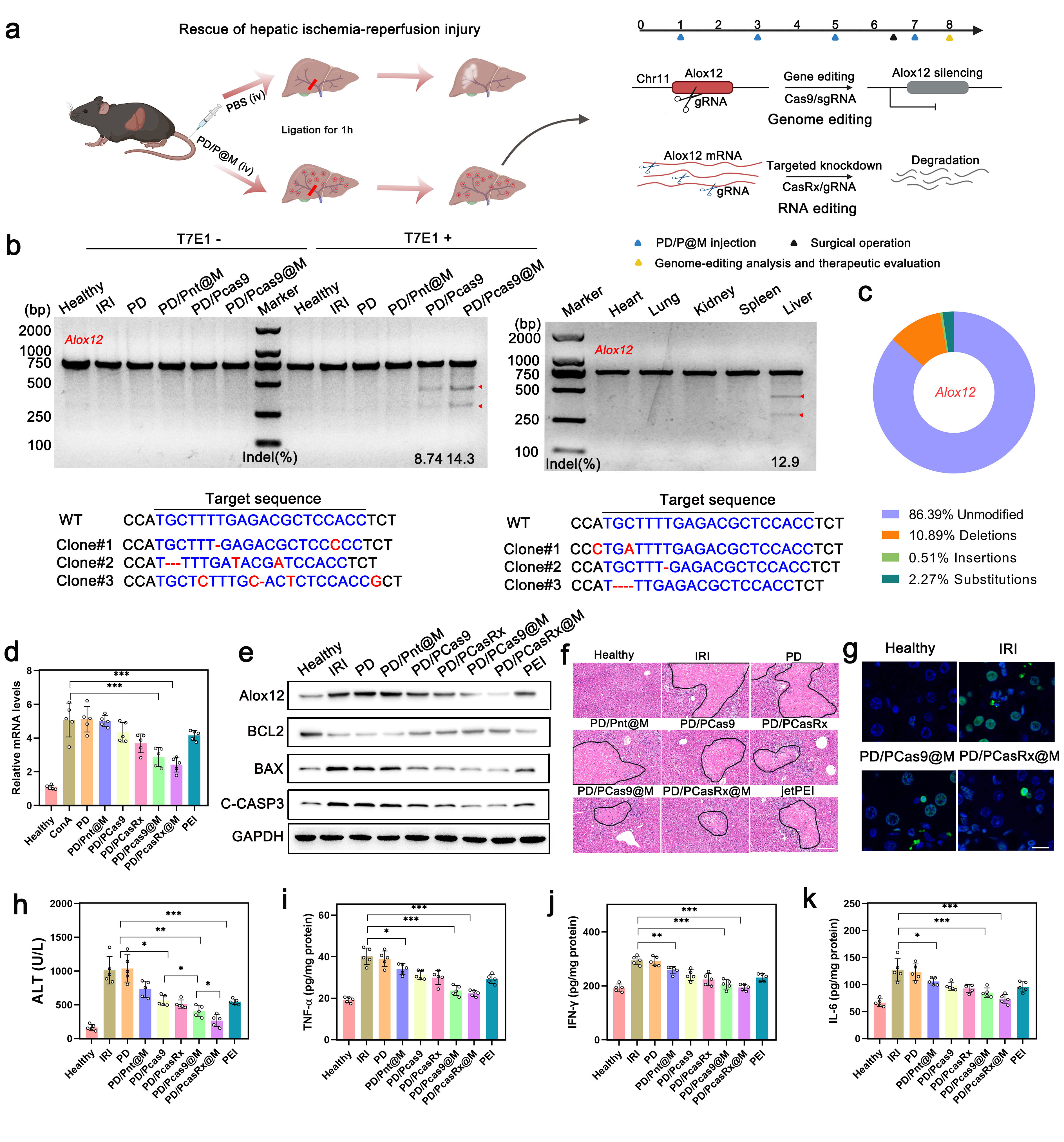


Fig. S3. PD/P@M mediated inflammatory genome editing for the protection of hepatic ischemia-reperfusion injury. (a) Schematic illustration of preventing hepatic ischemia-reperfusion injury by PD/P@M (created with BioRender.com). (b) The frequency of indel mutation of *Alox12* detected by T7E1 assay from the liver tissue after the indicated treatment (left) and representative Sanger sequencing results of T-A cloning after PD/Pcas9@M treatment (right). And indel mutations of *Alox12* locus detected by T7E1 assay (left) and Sanger sequencing from different organs after the treatment by PD/Pcas9@M. (c) Deep sequencing results of *Alox12* locus after PD/Pcas9@M-mediated transfection of P3-Cas9-GFP-U6-sgRNA plasmid in mice with hepatic ischemia-reperfusion (IR) injury. (d) The relative Alox12 mRNA levels in the livers of mice after the indicated groups. (e) Western blotting showing protein expression of Alox12, BCL2, BAX and C-CASP3 (cleaved caspase-3) in the liver of mice after the indicated treatment. (f) Hematoxylin-eosin (H&E) staining of the liver samples after the indicated treatment. In the H&E sections, the black dash line denoted the area of liver damage and tissue necrosis. Scale bar: 200 µm. (g) Terminal deoxynucleotidyl transferase dUTP nick-end labeling (TUNEL) staining of liver sections from IRI mice and indicated treatment groups. Scale bar = 20 μm. (h) The serum ALT (m) levels after the indicated treatment. (i) The protein level of tumor necrosis factor-α (TNF-α) and (j) interferon gamma (IFN-γ) analyzed by ELISA assay. (k) The protein level of interleukin-6 (IL-6) analyzed by ELISA assay. All the date were analyzed by One-way ANOVA with a Tukey’s post-hoc test. Mean ± S.D., n = 5. *P < 0.05; **P < 0.01 ***P < 0.001.


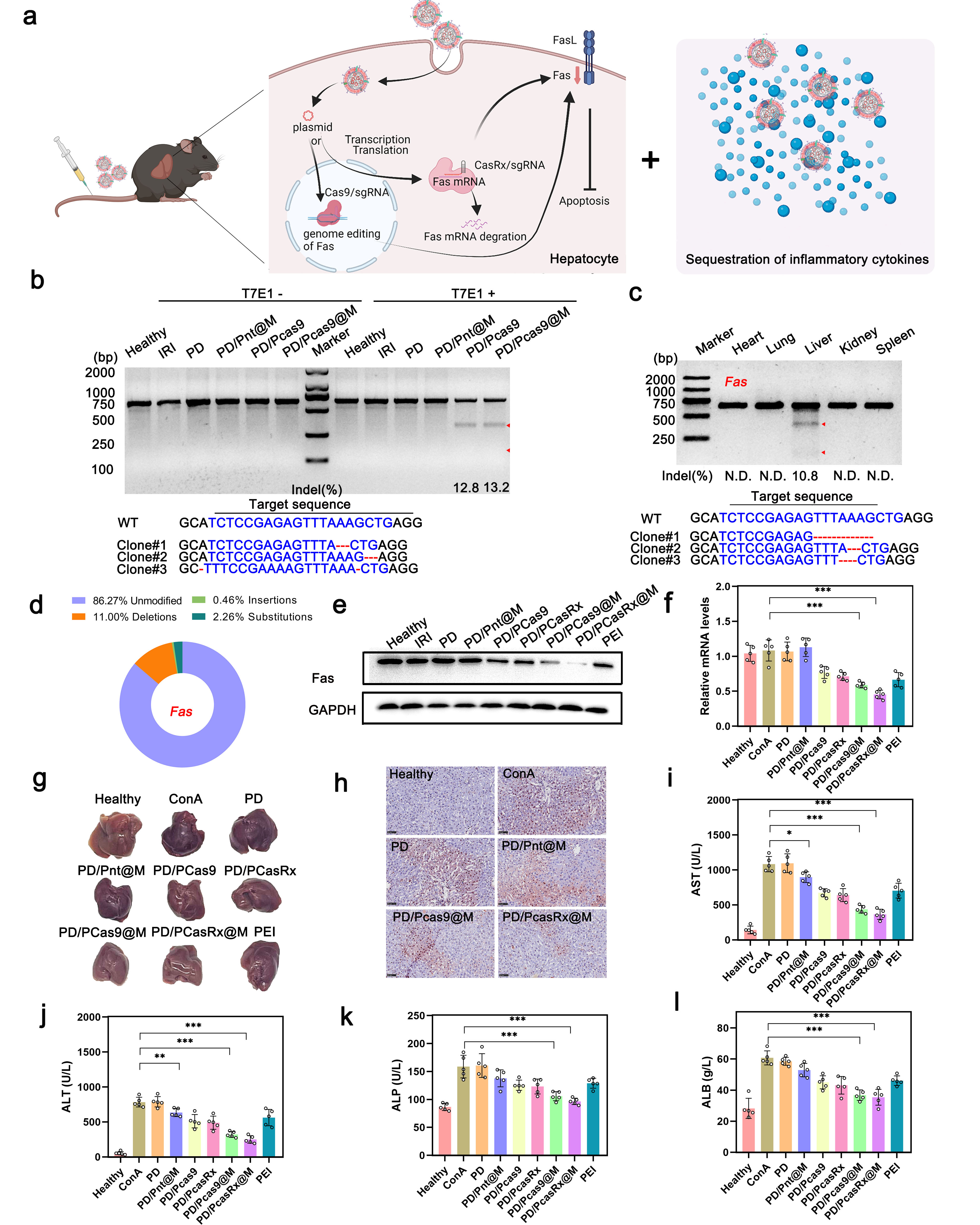


Fig. S4. *Fas* knockdown *in vivo* to rescue mice from acute liver injury. (a) Schematic illustration of *in vivo* delivery of Cas9 or CasRx plasmids enabled by PD/P@M for the treatment of ConA-induced hepatic injury (Created by BioRender.com). (b) Indel frequency of *Fas* validated by T7E1 assay (upper) and Sanger sequencing results (lower) in the liver of mice after the indicated treatment. (c) Genomic disruption of *Fas* after the treatment by PD/PCas9@M as analyzed by T7E1 in the different organs, and the representative Sanger sequencing of T-A cloning from the liver tissue. (d) Deep sequencing analysis of mutation frequency at *Fas* locus in the liver of mice after the treatment with PD/PCas9@M. (e) Western blotting analysis of the protein expression of *Fas* in the livers of mice with acute liver failure after indicated treatments. (f) Relative *Fas* mRNA level in the liver of mice after the indicated treatment. (g) The images of the liver from mice with acute liver injury after the indicated treatment. (h) Terminal deoxynucleotidyl transferase dUTP nick-end labeling (TUNEL) staining of liver sections from mice with acute liver failure after indicated treatments. Scale bar = 50 μm. The serum AST (i), ALT (j), ALP(k), and ALB (l) level after the treatment. All the date were analyzed by One-way ANOVA with a Tukey’s post-hoc test. Mean ± S.D., n = 5. *P < 0.05; **P < 0.01 ***P < 0.001.


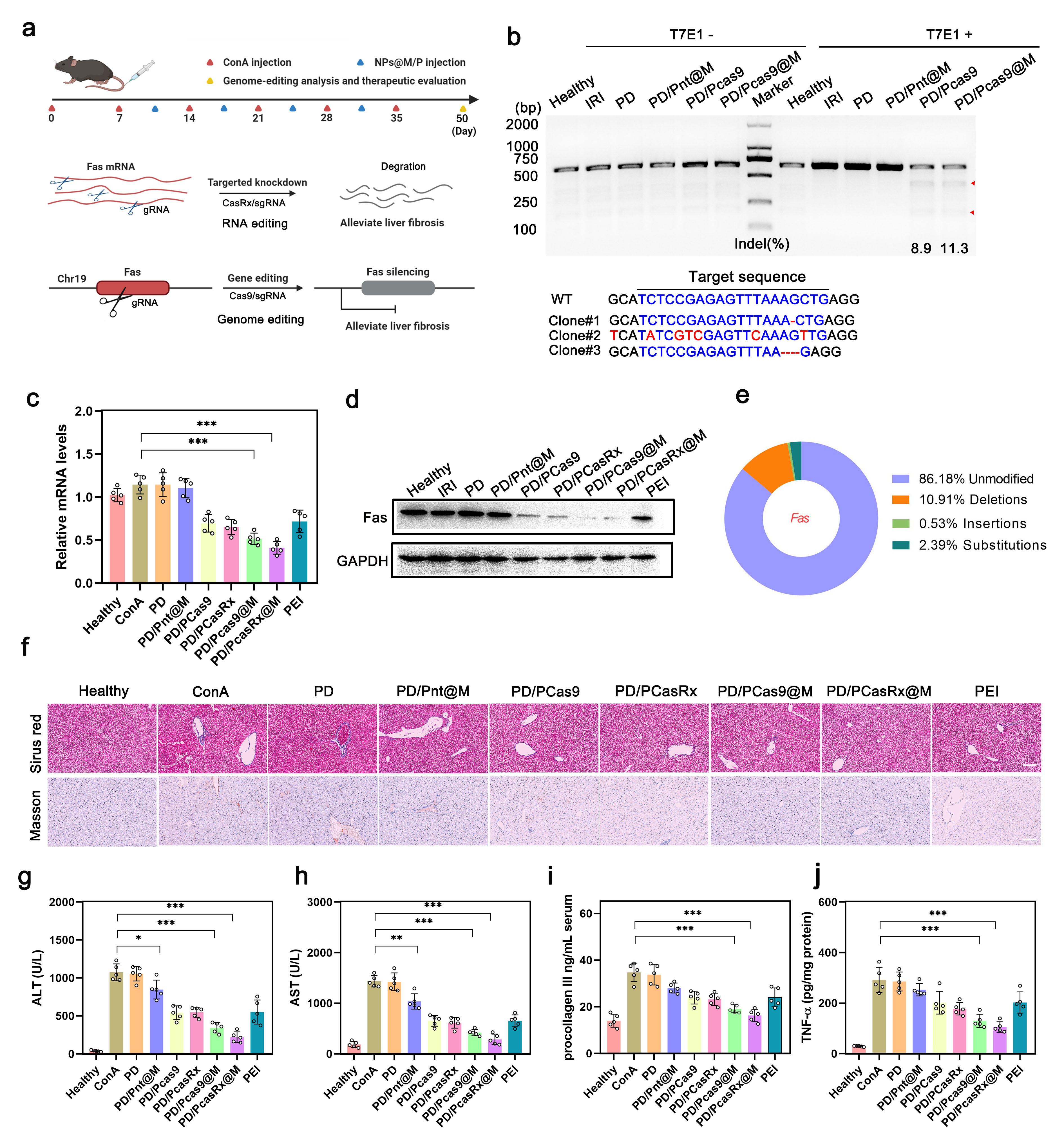


Fig. S5. Treatment of the mice with liver fibrosis by *Fas* knockdown *in vivo*. (a) Schematic illustration of PD/PCas9@M or PD/PCasRx@M-mediated treatment of hepatic fibrosis. ConA and PD/PCas9@M or PD/PCasRx@M complexes were systemically administered by tail vein (Created by BioRender.com). (b) Indel frequency of *Fas* in the liver T7E1 assay after the indicated treatment and Sanger sequencing result after indicated treatment. (c) Relative *Fas* mRNA level in the liver of mice after the indicated treatment. (d) Western blotting analysis of the protein expression of *Fas* in the livers of mice with liver fibrosis after indicated treatments. (e) Deep sequencing analysis of mutation frequency at *Fas* locus in the liver of mice after the treatment with PD/PCas9@M. (f) Liver sections were stained for masson and α-SMA to assess the degree of collagen deposition, fibrillar collagen, and activation of HSCs, respectively. Scale bar: 200 µm. The serum ALT levels (g) and AST levels (h) in mice with liver fibrosis after different treatments. All of the data analyzes as mean ± S.D., n = 5. (i) The serum procollagen type III (u) from the liver fibrosis mice administered with PD/PCas9@M or PD/PCasRx@M complexes. (j) The level of TNF-α in serum of mice after the indicated treatment. For (a) to (j), the abbreviation denotes the following: ConA, the treatment with concanavalin A; PD/Pnt@M, the complex of PD/P3-Cas9 plasmid with macrophage membrane for non-sense targeting; PD/PCas9@M, the complex of PD/P3-Cas9 plasmid with macrophage membrane for *Fas* targeting; PD/PCasRx@M, the complex of PD/P3-CasRx plasmid with macrophage membrane for *Fas* targeting. All of the data analyzes as mean ± S.D., n = 5. One-way ANOVA with a Tukey’s post-hoc test. *P < 0.05; **P < 0.01 ***P < 0.001.


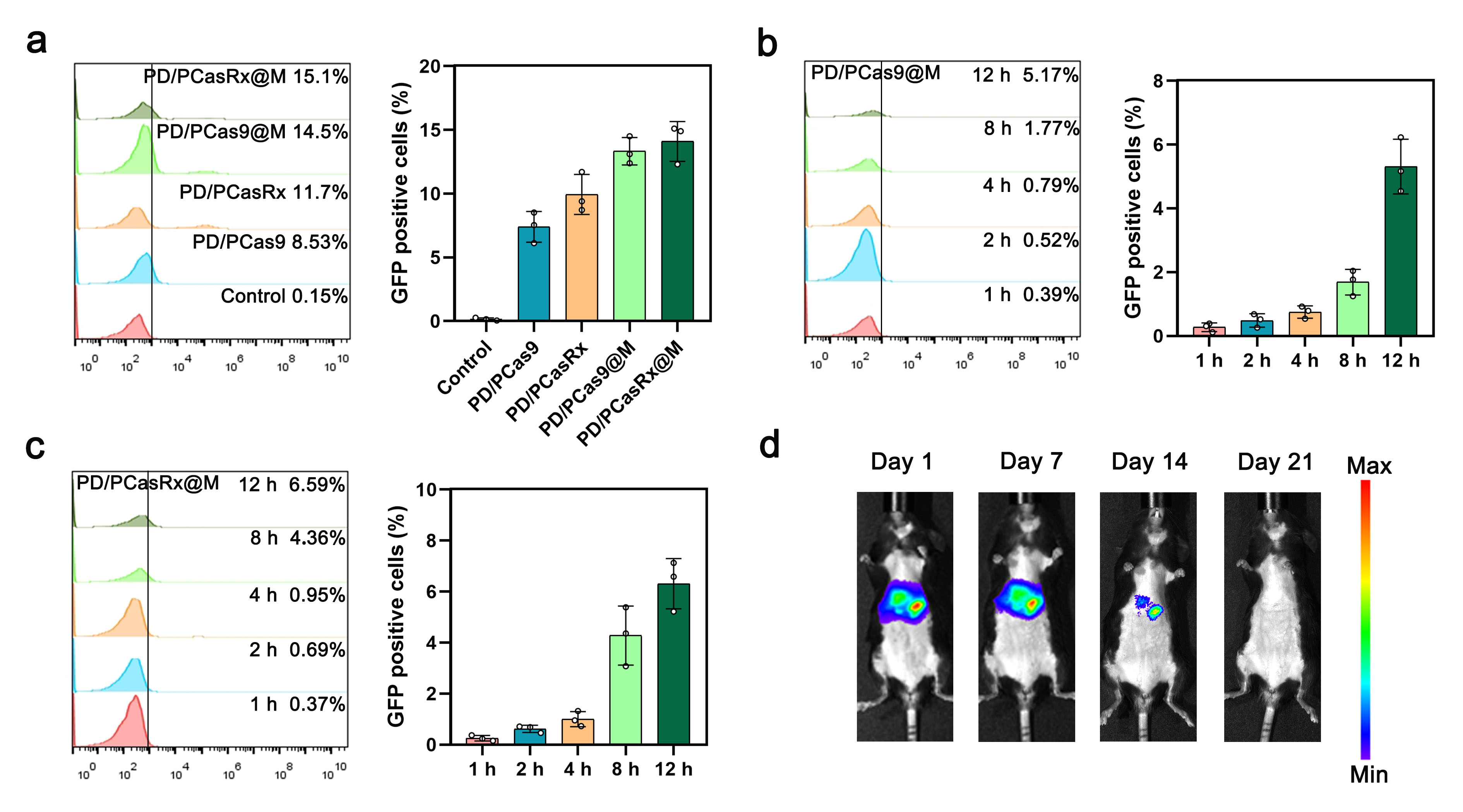


Fig S6. (a) GFP-positive cells in liver tissues were determined by flow cytometry after the systemic transfection of Cas9 or CasRX plasmid simultaneously encoding a GFP tag. (b) GFP-positive cells in liver tissues were determined by flow cytometry after the systemic injection of PD/PCas9@M at the indicated timepoint. (c) GFP-positive cells in liver tissues were determined by flow cytometry after the systemic injection of PD/PCasRx@M at the indicated timepoint. (d) In vivo luciferase expression in the liver tissue. The mice with liver fibrosis were systematically injected with PD/PCasRx@M complexes, in which the plasmid was simultaneously encoding luciferase. The fluorescence was detected at the indicated timepoint.


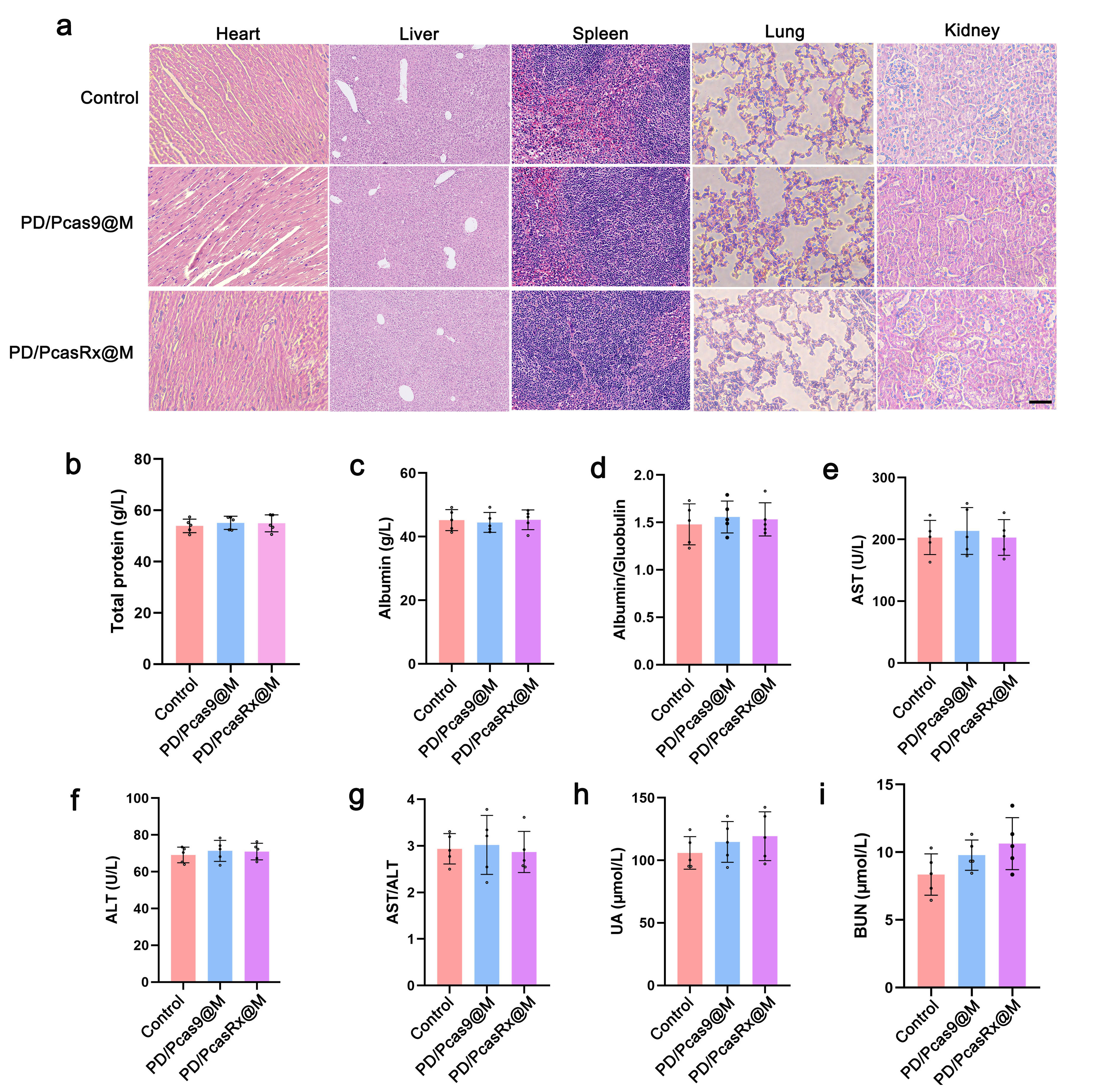


Fig. S7. Toxicity evaluation after treatment with PD/Pcas9@M or PD/PCasRx@M in normal mice. (a) The tissue slices from major organs were analyzed by H&E staining 28 days after the treatment. Scale bar = 50 μm. The levels of total protein (b), albumin (c), albumin/globulin (d), ALT (alanine aminotransferase) (e), AST (aspartate aminotransferase) (f), AST/ALT (g), UA (uric acid) (h), and BUN (blood urea nitrogen) (i) in serum at day 28. Data represent mean ± S.D. (n = 5)


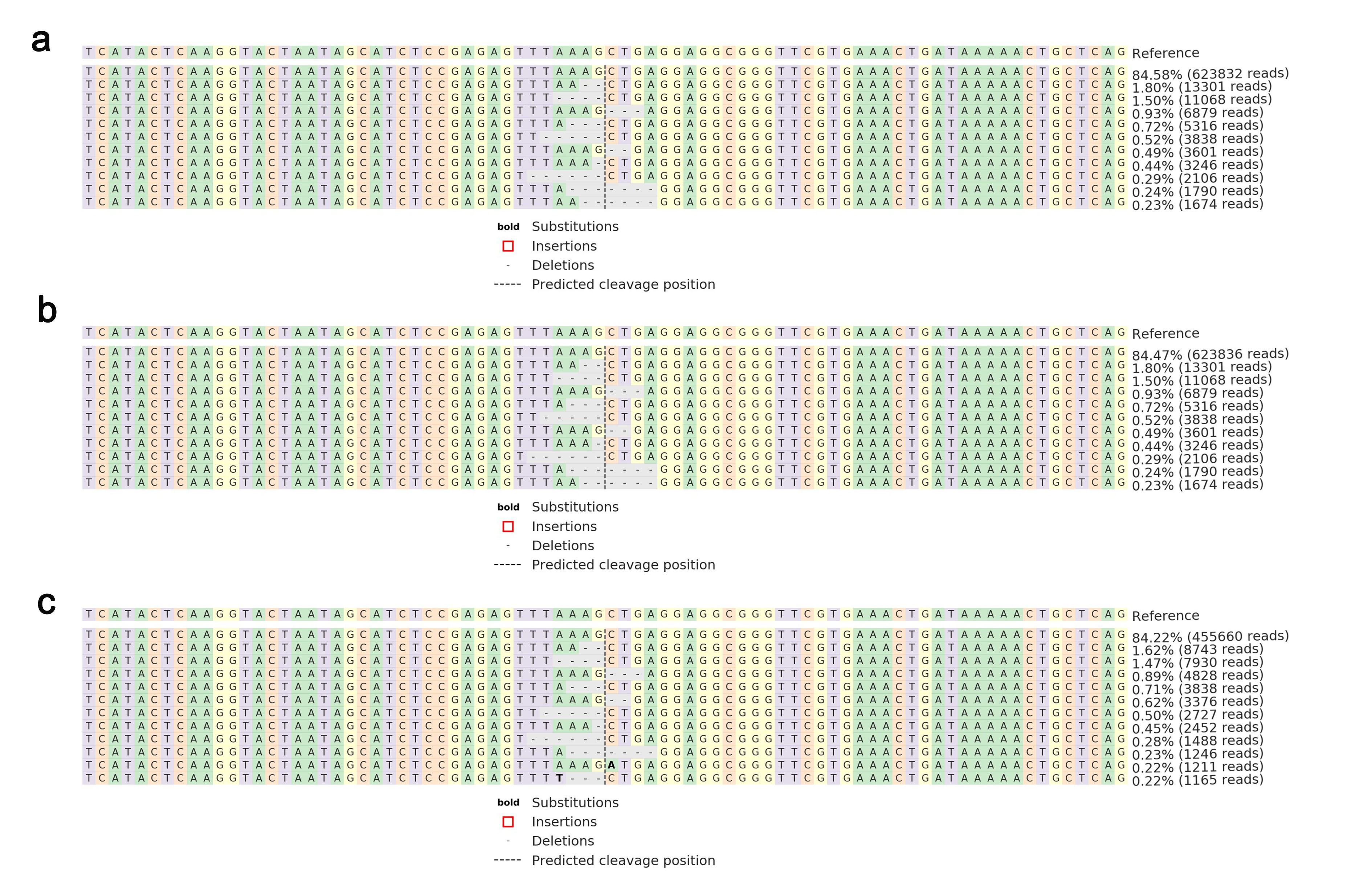


Fig. S8. Deep sequencing results of Alox12 locus after PD/Pcas9@M-mediated transfection of P3-Cas9-GFP-U6-sgRNA plasmid in mice with hepatic ischemia-reperfusion (IR) injury (a), acute liver injury (b), and liver fibrosis (c).

P3 promoter sequence (5’-3’)

tcgacACCGGCGCGCCGGGGGAGGCTGCTGGTGAATATTAACCAAGGTCACCCCAGTTATCGGAGGAGCAAACAGGGGCTAAGTCCACACGCGTGGTACCGTCTGTCTGCACATTTCGTAGAGCGAGTGTTCCGATACTCTAATCTCCCTAGGCAAGGTTCATATTTGTGTAGGTTACTTATTCTCCTTTTGTTGACTAAGTCAATAATCAGAATCAGCAGGTTTGGAGTCAGCTTGGCAGGGATCAGCAGCCTGGGTTGGAAGGAGGGGGTATAAAAGCCCCTTCACCAGGAGAAGCCGTCACACAGATCCACAAGCTCCTGgaatt

Fig S9. The sequence of P3 promoter.

Table S1. Primer sequences for sgRNA (Cas9) synthesis used in this study.

| **Primer names** | **Sequences (5’-3’)** | **Notes** |
| --- | --- | --- |
| sgEMX1-F | CACCGAGTCCGAGCAGAAGAAGAA | *EMX1* targeting |
| sgEMX1-R | AAACTTCTTCTTCTGCTCGGACTC |  |
| sgAlox12-F | CACCGGGTGGAGCGTCTCAAAAGCA | *Alox12* Targeting |
| sgAlox12-R | AAACTGCTTTTGAGACGCTCCACCC |  |
| sgFas-F | CACCGTCTCCGAGAGTTTAAAGCTG | Fas Targeting |
| sgFas-R | AAACCAGCTTTAAACTCTCGGAGAC |  |

Table S2. Primer sequences for sgRNA (CasRx) synthesis used in this study.

| **Primer names** | **Sequences (5’-3’)** | **Notes** |
| --- | --- | --- |
| sgAlox12-1-F | AAACCAGAGAGGTGGAGCGTCTCAAAAGCATT | *Alox12* targeting |
| sgAlox12-1-R | AAAAAATGCTTTTGAGACGCTCCACCTCTCTG |  |
| sgAlox12-2-F | AAACGCAGTTGGAAATCTGAATTTCGGACCCT | *Alox12* Targeting |
| sgAlox12-2-R | AAAAAGGGTCCGAAATTCAGATTTCCAACTGC |  |
| sgAlox12-3-F | AAACTTGAAATTGGCTGAGCACAGCTTTGGTT | Alox12 Targeting |
| sgAlox12-3-R | AAAAAACCAAAGCTGTGCTCAGCCAATTTCAA |  |
| sgFas-1-F | AAACTGTCCATGTACTCCTTCCCTTCTGTGCT | Fas Targeting |
| sgFas-1-R | AAAAAGCACAGAAGGGAAGGAGTACATGGACA |  |
| sgFas-2-F | AAACGCACGGCTCAAGGGTTCCATGTTCACAT | Fas Targeting |
| sgFas-2-R | AAAAATGTGAACATGGAACCCTTGAGCCGTGC |  |
| sgFas-3-F | AAACTTCTGCTCAGCTGTGTCTTGGATGCTGT | Fas Targeting |
| sgFas-3-R | AAAAACAGCATCCAAGACACAGCTGAGCAGAA |  |

Table S3. Primer sequences for PCR amplification of target genes.

| **Primer names** | **Sequences (5’-3’)** |
| --- | --- |
| EMX1-F | ATGTTAGACCCATGGGAGCAG |
| EMX1-R | GATTGGAGACACGGAGAGCAG |
| Alox12-F | CCCTAGTGGCCCCTTTTCATTA |
| Alox12-R | TCTATCTCCCTGCACCATGAC |
| Fas-F | CTCATCTCCTGGCAACCACC |
| Fas-R | ATGGTGAGCTTTGAGATGCCT |

Table S4. Primer sequences for Q-PCR analysis of target genes.

| **Nucleic Acid ID** | **Sequences (5’-3’)** |
| --- | --- |
| Alox12-F | GTTCCACACATCCGTTACACT |
| Alox12-R | CCGAGTAAGCAACTGAACATGG |
| Fas-F | TATCAAGGAGGCCCATTTTGC |
| Fas-R | TGTTTCCACTTCTAAACCATGCT |
| GAPDH-F | AGGTCGGTGTGAACGGATTTG |
| GAPDH-R | TGTAGACCATGTAGTTGAGGTCA |
